# Aberrant neural synchrony in the maternal immune activation model: using translatable measures to explore targeted interventions

**DOI:** 10.3389/fnbeh.2013.00217

**Published:** 2013-12-27

**Authors:** Desiree D. Dickerson, David K. Bilkey

**Affiliations:** ^1^IST AustriaKlosterneuburg, Austria; ^2^Department of Psychology, University of OtagoDunedin, New Zealand

**Keywords:** schizophrenia, animal model, Poly I:C, developmental, environmental risk, prenatal infection, synchrony

## Abstract

Maternal exposure to infection occurring mid-gestation produces a three-fold increase in the risk of schizophrenia in the offspring. The critical initiating factor appears to be the maternal immune activation (MIA) that follows infection. This process can be induced in rodents by exposure of pregnant dams to the viral mimic Poly I:C, which triggers an immune response that results in structural, functional, behavioral, and electrophysiological phenotypes in the adult offspring that model those seen in schizophrenia. We used this model to explore the role of synchronization in brain neural networks, a process thought to be dysfunctional in schizophrenia and previously associated with positive, negative, and cognitive symptoms of schizophrenia. Exposure of pregnant dams to Poly I:C on GD15 produced an impairment in long-range neural synchrony in adult offspring between two regions implicated in schizophrenia pathology; the hippocampus and the medial prefrontal cortex (mPFC). This reduction in synchrony was ameliorated by acute doses of the antipsychotic clozapine. MIA animals have previously been shown to have impaired pre-pulse inhibition (PPI), a gold-standard measure of schizophrenia-like deficits in animal models. Our data showed that deficits in synchrony were positively correlated with the impairments in PPI. Subsequent analysis of LFP activity during the PPI response also showed that reduced coupling between the mPFC and the hippocampus following processing of the pre-pulse was associated with reduced PPI. The ability of the MIA intervention to model neurodevelopmental aspects of schizophrenia pathology provides a useful platform from which to investigate the ontogeny of aberrant synchronous processes. Further, the way in which the model expresses translatable deficits such as aberrant synchrony and reduced PPI will allow researchers to explore novel intervention strategies targeted to these changes.

## Introduction

Schizophrenia is a neurodevelopmental disorder characterized by a number of heterogeneous symptoms that include cognitive deficits. The aetiology, neuropathology, and response to pharmacotherapy show considerable variability across the population making it particularly difficult to determine the underlying causal mechanisms. Despite this heterogeneity, several consistent findings have emerged from the literature. First, it is clear that both genetic and environmental factors contribute to the aetiology of schizophrenia, and second, it is also apparent that the impact of these factors is time-dependent, with greater vulnerability occurring during critical neurodevelopmental windows.

A number of investigations have searched for an overarching mechanism that might underpin the diverse range of deficits described in schizophrenia and that would also account for the neurodevelopmental factors that have been implicated in its aetiology. Recent research has focussed on the role of temporal coordination of neural activity. In this work, it has been proposed that a temporal synchronization process normally functions as a binding mechanism that supports the integration of neural processing that occurs across widespread areas of the brain (Von Der Malsburg, 1985; Singer, 1999; Buzsaki et al., 2004). It is hypothesized that this synchronization mechanism is disrupted in schizophrenia (Uhlhaas and Singer, 2006). A considerable body of data supports this proposal. For example, a disruption in synchronized neural activity, within both local and long-range networks, has been reported in individuals with schizophrenia. This aberrant network coupling has been associated with some of the prominent positive, negative, and cognitive symptoms of the disorder (Cho et al., 2006; Uhlhaas and Singer, 2006; Pachou et al., 2008; Spencer et al., 2009) and is present in first-episode patients (Koenig et al., 2001; Symond et al., 2005; Williams et al., 2009a,b) as well as in individuals at high-risk of presenting with the disorder (Mann et al., 1997; Winterer et al., 2003a,b; Hong et al., 2004a; Donkers et al., 2011; Hall et al., 2011). Dysfunctional synchronization of neural networks may, therefore, degrade the processes by which information is integrated, and in doing so generate at least some of the constellation of deficits that characterize the symptomatology.

To date, exploration of aberrant network coupling in schizophrenia has largely centered on data obtained from EEG and MEG recordings conducted within the human patient population. Although these studies are valuable, the recent use of animal models of schizophrenia that exhibit disordered synchrony has provided a means to investigate the mechanisms that underlie the disorder with greater spatial and temporal resolution of the phenomenon and better access to underlying physiology (Uhlhaas, 2013).

## The MIA model of schizophrenia

Although modeling a complex human disorder such as schizophrenia in its entirety is not possible, a variety of animal models have been developed in order to address specific hypotheses regarding the aetiology, neuropathophysiology, and symptomatology of schizophrenia from a neurodevelopmental perspective. It is known that environmental insults that occur during critical periods of brain development, such as during the prenatal period, are associated with long-lasting changes in brain development and function. These changes can result in subsequent adult neuropathology (Rees and Inder, 2005) and specifically an increased risk of schizophrenia in the offspring (Weinberger, 1987; Mednick et al., 1988; Brown and Derkits, 2010). Maternal infection, such as influenza, occurring during pregnancy, is one such insult. This phenomenon was first highlighted in a study that followed a population of children born after the 1957 Influenza epidemic in Finland (Mednick et al., 1988). It was determined that there was a 2- to 3-fold increased risk of schizophrenia in the offspring of women who were in mid-gestation during the epidemic. This finding was subsequently replicated in several studies (Barr et al., 1990; O'Callaghan et al., 1991; Sham et al., 1992; Mednick et al., 1994; Takei et al., 1996; Limosin et al., 2003; Selten et al., 2010) although a failure to find evidence for a relationship between prenatal infection and schizophrenia (Mino et al., 2000) has also been reported. This variability is most likely due to the generous exposure-to-infection classification used within some of these epidemiological studies – women simply had to be pregnant at the time of the epidemic (Brown and Derkits, 2010). Many women who were actually unexposed to the virus during pregnancy were likely to have been misclassified as exposed, biasing effect sizes toward zero (Brown and Derkits, 2010).

Subsequent studies have had access to maternal sera which has allowed for testing that established a more substantive link. Maternal sera studies determine a mother's exposure to infection by identifying antibodies to specific infectious agents that were present during pregnancy. Brown et al. (2004) conducted a breakthrough study that compared maternal serum samples from mothers of children who later developed schizophrenia with maternal serum from mothers whose children did not. The findings revealed a 3-fold increase in risk of developing schizophrenia in offspring from mothers with elevated levels of influenza antibodies during the first half of gestation. This rose to a 7-fold increase in risk where exposure to influenza was localized to the first trimester of gestation. This relationship was not apparent if exposure occurred in the second half of gestation. While the gestational period at which greatest risk was conferred differed between Brown et al. (2004), and several previous epidemiological findings e.g., (Mednick et al., 1988). Brown and colleagues (Brown et al., 2004) argued that the timing of exposure in the previous epidemiological findings was related to peak-periods of the epidemic, and not to the exposure of individual cases.

Influenza has been the most widely studied pathogen in the MIA literature, because of its prevalence in the general population. Other studies have, however, revealed that less prevalent infections and viruses including bacterial infections (Clarke et al., 2009; Sorensen et al., 2009), bronchopneumonia (Brown et al., 2000), polio (Suvisaari et al., 1999; Cahill et al., 2002), herpes simplex virus (Buka et al., 2001, 2008; Babulas et al., 2006), rubella (Brown et al., 2000, 2001), and toxoplasma gondii (Brown et al., 2005; Mortensen et al., 2007) are also associated with increased risk for schizophrenia in the offspring of those infected. This common effect across various infections suggests that a specific infection or virus does not, in and of itself, increase the risk of schizophrenia. Rather, it appears that the mother's immune response to the infectious agent plays a key role (Patterson, 2002).

The body produces a number of signaling molecules in response to infection. These cytokines, which are critically involved in the host's immune response, are a family of soluble polypeptides that are able to enter the placenta and are capable of permeating the blood brain barrier (Gilmore and Jarskog, 1997). These same cytokines are also critical signaling molecules that are normally involved in brain development (Cannon, 2000), but when pathologically elevated in response to maternal infection they can have adverse effects on the neurodevelopment of the offspring (Gilmore and Jarskog, 1997). These adverse effects on foetal brain development include changes in the proliferation, differentiation, apoptosis, neurite growth, and gene expression, of both neurons and glia (Mehler and Kessler, 1998; Patterson, 2002). While the precise pathological mechanisms remain unclear, several of the cytokines (e.g., IL-6, IL-8, & IL-10) produced in response to maternal infection have also been shown to be elevated in both first-episode schizophrenia and acute relapsed patients Potvin, 2008 #1902; Miller, 2011 #1903}.

The MIA model in rodents can be induced through maternal exposure to the synthetic double-stranded RNA analog, Poly I:C, during gestation. As with the double stranded RNA generated during a viral infection, this synthetic RNA is detected by the immune system as being foreign by the transmembrane protein toll-like receptor 3 TLR-3) (Alexopoulou et al., 2001). Binding to this pathogen recognising receptor, TLR-3, triggers the production and release of several pro-inflammatory cytokines including IL-1β, IL-6, IL-8, IL-10, and TNF-α (Fortier et al., 2004; Meyer et al., 2006b; Cunningham et al., 2007; Patterson, 2009; Hsiao and Patterson, 2011) and type I interferons (Fortier et al., 2004; Koga et al., 2009). Critically, these elevated cytokine levels are found in the placenta, amniotic fluid, and in the foetus, including the foetal brain (Meyer et al., 2006a, 2009; Jonakait, 2007). This acute response lasts for approximately 24–48 h depending on the dose (Meyer et al., 2005; Cunningham et al., 2007); providing a window that allows for the precise targeting of the intervention to different stages of foetal neurodevelopment (Meyer et al., 2005, 2006b).

Since the development of the Poly I:C-induced MIA model (Shi et al., 2003; Zuckerman et al., 2003), a number of studies have documented structural, neurochemical, behavioral and electrophysiological phenotypes consistent with schizophrenia-like pathology in both rats and mice. Morphological abnormalities have included evidence of reduced hippocampal volume (Piontkewitz et al., 2009) and the post-pubertal enlargement of the lateral and fourth ventricles in both mouse and rat MIA offspring (Li et al., 2009; Piontkewitz et al., 2009); mirroring two of the most consistent abnormalities observed in schizophrenia. While several studies have also revealed moderate to severe pyramidal cell loss in the CA1 region of the HPC in adult rat offspring (Zuckerman and Weiner, 2003; Zuckerman et al., 2003), others have reported no overall hippocampal cell loss or volume reduction (Meyer et al., 2006b; Oh-Nishi et al., 2010). Reduced myelination and decreased axonal size have been detected in the HPC during early postnatal development of MIA mouse offspring (Makinodan et al., 2008); changes that were no longer present at adulthood, suggesting a neurodevelopmental delay in myelination maturation (Makinodan et al., 2008). Adult neurogenesis in the sub-granular zone of the DG of the HPC has been shown to be disrupted, with reductions in the number of neuronal progenitor cells found in the dorsal DG of MIA mouse offspring (Meyer et al., 2006b; Meyer and Feldon, 2010).

Maternal immune activation (MIA) through Poly I:C has also been shown to produce neurochemical changes in the adult offspring that mimic some of the changes observed in schizophrenia. These include alterations in the mesocorticolimbic dopaminergic system; a system that may be the final common pathway in schizophrenia pathology (Lodge and Grace, 2011; Eyles et al., 2012). Winter et al. (2009) noted a significant reduction in the dopamine metabolite homovanillic acid in the nucleus accumbens, while (Winter et al., 2009) (Zuckerman et al., 2003) reported no changes in basal striatal dopamine levels in adult animals that had received prenatal Poly I:C exposure. Sub-chronic administration of Poly I:C from GD12—GD17, was found to induce dopamine hyperfunction and decreased D2-like receptor binding in the striatum of adult MIA mice (Ozawa et al., 2006). It has also been shown that levels of Tyrosine hydroxylase (TH), the rate-limiting enzyme of dopamine/noradrenalin synthesis *in vivo*, is enhanced in the striatum (Meyer et al., 2008a) and the number of TH-positive dopamine neurons was shown to be increased in the substantia nigra and ventral tegmental area in both foetal (GD19) and adult MIA offspring (Vuillermot et al., 2010). Changes to the dopamine system in the PFC have also been noted in adult MIA offspring, including increased basal levels of dopamine (Winter et al., 2009) and long-lasting reductions in dopamine D1 and D2 receptor immunoreactivity in the medial PFC (Meyer et al., 2008a,b).

GABAergic dysfunction is one of the most consistent findings in the schizophrenia literature (Akbarian and Huang, 2006). Since GABAergic interneurons are known to play a prominent role in synchronous oscillatory activity (Bartos et al., 2007) they have been implicated in the dysfunction of neural synchrony observed in individuals with schizophrenia. The ability of the MIA model to mimic pathologically relevant disruptions in GABAergic neurotransmission is therefore likely to be critical for any attempts to examine the “synchrony hypothesis” in an animal model. Presynaptic GABAergic dysfunction is evident in the MIA model and expressed as a reduced number of GABAergic Reelin- and Parvalbumin-positive cells in the PFC of offspring following both mid- and late-gestational exposure to MIA (Meyer et al., 2008b). A reduced number of PV-positive cells has also been reported in the ventral HPC, specifically following late-gestational MIA exposure (Meyer et al., 2008b) with reduced Reelin-positive cells in dorsal CA1 subfield of the HPC following mid-gestational MIA exposure (Meyer et al., 2006b). Post-synaptic GABAergic dysfunction was also noted, with GABA_A_ receptor α2 immunoreactivity shown to be up-regulated in the ventral DG and basolateral amygdala of mid-gestational MIA exposed offspring (Nyffeler et al., 2006; Meyer et al., 2008b).

Abnormalities in glutamatergic transmission may also play a role schizophrenia, either directly or through an influence on GABAergic function (Schwartz et al., 2012). Increased basal levels of extracellular glutamate in the PFC (Roenker et al., 2011) and HPC (Ibi et al., 2009) occur in adult MIA rat offspring. Thus exposure to MIA in utero alters several neurotransmitter systems known to be disrupted in schizophrenia, including those using dopamine, glutamate, GABA and serotonin systems (Winter et al., 2009; Bitanihirwe et al., 2010). As noted, a number of these neurotransmitter changes are found to occur in the HPC and PFC, consistent with changes in schizophrenia.

A notable and consistently observed feature of schizophrenia is a reduction in sensorimotor gating. It can be measured in humans and other animals by testing the degree of pre-pulse inhibition (PPI) of an acoustic startle. This procedure measures the ability of a weak pre-stimulus to reduce the startle response to a loud acoustic stimulus (Braff et al., 2001a). Deficits in PPI of the acoustic startle response are a stable cross-species phenomenon that is used as a hallmark measure of schizotypal behavioral deficits in animal models of schizophrenia. Reductions in PPI have repeatedly been shown in the MIA model, across both mice and rats, across varying doses and gestational timing of the insult (Meyer et al., 2006b, 2008b; Nyffeler et al., 2006; Ozawa et al., 2006; Smith et al., 2007; Wolff and Bilkey, 2008; Shi et al., 2009). In mice, PPI deficits present with a post-pubertal onset (Ozawa et al., 2006; Vuillermot et al., 2010), and the deficit has been shown by our lab to be present in both juvenile (Wolff and Bilkey, 2010) and adult rats (Wolff and Bilkey, 2008); a finding that is consistent with data showing PPI deficits in both prodromal presentation (Quednow et al., 2008) and individuals at high-risk for schizophrenia (Cadenhead et al., 2000; Kumari et al., 2008).

A further deficit in information processing that may contribute to some of the symptoms of schizophrenia centers around the phenomenon of latent inhibition (LI) (Weiner, 2003). LI is a measure of the ability to ignore irrelevant stimuli and it is disrupted in adult MIA, but not post-pubertal, rat offspring (Zuckerman and Weiner, 2003; Zuckerman et al., 2003; Smith et al., 2007) as well as in adult mice following early to mid-gestational (Meyer et al., 2006c) and late gestational exposure (Bitanihirwe et al., 2010). Memory deficits have also been reported in the MIA model, including those observed in novel object recognition (Ozawa et al., 2006; Wolff et al., 2011) and the Morris Water Maze task (Meyer et al., 2008b; Savanthrapadian et al., 2013). An increased level of anxiety is also indicated through reduced open-field and novel object exploration (Meyer et al., 2006b; Ozawa et al., 2006; Smith et al., 2007); as is increased perseveration, reflected in impaired reversal learning in MIA exposed offspring (Meyer et al., 2006b). Amphetamine—(dopamine receptor agonist) and MK-801—(a non-competitive NMDA receptor antagonist) induced increases in locomotion also occur with post-pubertal onset (Zuckerman and Weiner, 2003, 2005; Ozawa et al., 2006; Meyer et al., 2008b). This elevated locomotor response to MK-801 has been attributed to a hyper-responsive dopamine system (Roenker et al., 2011).

Recent data indicate that the MIA intervention may also model the “two-hit” hypothesis of schizophrenia which proposes that genetic or environmental factors (the first “hit”) generate a vulnerability to schizophrenia which may then be triggered by a second “hit,” for example, stress or drug use, that occurs during early adulthood (Giovanoli et al., 2013) demonstrated that a low-dose Poly I:C intervention, when followed with a stressor applied in the peri-pubertal period, generated schizophrenia-like changes in the adult animals. Neither of these interventions on their own produced a response, indicating that some synergy was occurring between the two “hits,” as has been hypothesized to occur in the disease.

In summary, while prenatal infection accounts for a relatively small proportion of the risk for developing schizophrenia, the fact that the MIA model induces so many features of a schizophrenia-like phenotype attests to the potential impact of prenatal factors on the developing foetus and its subsequent pathology. The MIA model induces structural, neurochemical, electrophysiological, pharmacological, and behavioral deficits that are consistent with a schizophrenia-like presentation and its neurodevelopmental origins. The MIA model provides, therefore, a platform from which to explore not only putative neuropathological underpinnings of symptoms and deficits, but possible intervention strategies as well. Furthermore, it allows for the targeting of critical windows in neurodevelopment in order to test many schizophrenia-relevant hypotheses.

## Synchronization

Schizophrenia has long been described as a disruption in integrative processes; a “loss of inner unity of the activities of intellect, emotion, and volition”, and an impaired “intrapsychic coordination” (Bleuler, 1911; Kraepelin, 1919). More recent models have similarly proposed that an underlying failure to functionally integrate neural systems occurs within the brain during schizophrenia; a concept captured by the “disconnection hypothesis” (Friston, 1998; Phillips and Silverstein, 2003). In this vein, it has been proposed that temporal synchronization of neural network activity provides the binding mechanism that normally facilitates the integration of diverse neural processes within the brain (Singer, 1999; Buzsaki and Draguhn, 2004) and that in schizophrenia, these synchronization processes are disrupted (Cho et al., 2006; Uhlhaas et al., 2006; Pachou et al., 2008; Spencer et al., 2009; Uhlhaas and Singer, 2010, 2011, 2012; Uhlhaas, 2013).

To date, the evidence in support of this hypothesis has indicated that both local and distal neuronal network communication is disrupted in schizophrenia and that these disruptions are linked to functional impairments and/or symptom presentation. Aberrant synchrony has been associated with perceptual impairments (Spencer et al., 2003, 2004; Lee et al., 2010; Spencer, 2011; Comparelli et al., 2013) and with deficits in working memory (Schmiedt et al., 2005; Basar-Eroglu et al., 2007; Pachou et al., 2008; Haenschel et al., 2009; Barr et al., 2010) and cognitive control (Cho et al., 2006). Deficits in synchrony have also been shown during resting-state and during sleep. Indeed, sleep is consistently found to be affected in schizophrenia patients. Correspondingly, sleep spindles—bursts of 7–14 Hz oscillatory activity, thought to maintain the stability of sleep—have been shown to be reduced in frequency, duration, and amplitude in schizophrenia (Ferrarelli et al., 2007, 2010; Manoach et al., 2010; Keshavan et al., 2011). These aberrant spindles were also found to correlate with impaired memory recall following sleep (Manoach et al., 2010; Wamsley et al., 2012).

These deficits in synchrony have been reported to occur in both chronic patients and unmedicated individuals e.g., (Gallinat et al., 2004), as well as in first-episode patients (Koenig et al., 2001; Symond et al., 2005; Williams et al., 2009a,b), suggesting that it is unlikely to be simply an artifact of medication, illness chronicity and subsequent illness heterogeneity, and/or institutionalization. This gives credence to the theory that aberrant temporal connectivity may be a fundamental, trait-like feature of this disorder. Moreover, healthy individuals with an elevated genetic risk for the disorder have also been shown to possess qualitatively similar alterations in synchronous neural activity; although to a lesser degree than individuals diagnosed with schizophrenia (Mann et al., 1997; Winterer et al., 2001, 2003a; Hong et al., 2004a,b; Donkers et al., 2011; Hall et al., 2011). Such findings argue that disrupted synchrony may represent a neurobiological endophenotype for heritability of the disorder (Hall et al., 2011).

The finding that aberrant synchronous network activity appears to be present at disease onset and is found in those with increased genetic risk is consistent with the neurodevelopmental hypothesis. It suggests that alterations in synchrony may represent a premorbid state that may be due to disrupted developmental processes, rather than a consequence of a current or previous schizophrenic episode. Indeed, during healthy brain development, neural networks, and connectivity undergo considerable change in structure and function during the transition period from adolescence to adulthood. Such brain changes include GABAergic (Hashimoto et al., 2009; Fung et al., 2010) and dopaminergic maturation (O'Donnell, 2010), reduction in gray matter volume (Giedd et al., 2009) and protracted white matter maturation (Benes, 1989; Benes et al., 1994; Cunningham et al., 2002; Cressman et al., 2010). The oscillatory activity of neural networks is, perhaps not surprisingly, also found to undergo periods of change during this late phase of maturation preceding adulthood (Uhlhaas et al., 2009; Uhlhaas and Singer, 2011). Notably, this late maturation phase corresponds to the onset of several pathological brain states including the characteristic post-pubertal onset of schizophrenia (Uhlhaas et al., 2009; Uhlhaas and Singer, 2011).

## HPC—mPFC communication

The HPC and PFC are two brain regions that are anatomically separated, and yet are required to communicate during various tasks requiring high-order cognitive processing. For example, performance on a spatial learning task is impaired when the HPC-mPFC pathway is disrupted (Floresco et al., 1997) and both appear to play a role in the consolidation of learning, such that the HPC makes a dominant contribution during the early phases of learning while the PFC appears to play a more considerable role during later stages of learning and consolidation (Bontempi et al., 1999; Frankland et al., 2004; Maviel et al., 2004). Lesion studies involving these two regions support the evidence for an early and late stage and the dichotomous contribution of each region to this process (Takehara et al., 2003).

Furthermore, the dorsal HPC and the mPFC have consistently been shown to temporally coordinate their activity during a variety of behavioral spatial tasks. (Siapas et al., 2005) was the first to demonstrate that neurons in the mPFC phase-locked their firing to theta oscillations recorded in the HPC LFP in freely behaving rats. This phase-locking was most prominent when the HPC theta oscillation was delayed by 50 ms, suggesting a directionality to the association in which HPC activity precedes PFC activity. Hyman et al. (2005) similarly showed that mPFC cells were modulated by theta oscillations recorded in the HPC, and that they shifted between phasic and non-phasic relationships depending on behavior (e.g., movement direction on a linear track). While neither of these studies demonstrated a functional role for this coupling, subsequent studies have demonstrated a behaviorally relevant temporal coordination between HPC—mPFC network activity. Jones and Wilson (2005b) simultaneously recorded single-unit activity in both the HPC and mPFC while the rat performed a spatial memory task. They too show that firing between the HPC and mPFC is synchronized, and further, that synchrony between these regions is greatest at the decision-making point of the spatial working memory task. They went on to show that, in response to this HPC entrainment, mPFC neuronal firing exhibited a theta phase precession effect similar to that of HPC neurons that increased alongside phase-locking at the choice point in the task (Jones and Wilson, 2005a). These oscillatory coupling mechanisms may serve to integrate the relative contributions of each region, the HPC involvement in spatial working memory and the mPFC role in decision-making, necessary for the rats performance in this spatial memory task (Hyman et al., 2011).

Building on this initial cluster of findings, Benchenane et al. (2010) have shown that LFP recorded in both the HPC and mPFC during a Y maze decision-making task are strongly coherent in the theta frequency band. Further, coherence between these key regions peaked at the choice point within the decision-making task. Coherence also increased as the animal's performance improved over time, and, on average, higher coherence predicted higher performance and low coherence predicted poorer performance. Hyman et al. (2010) also demonstrated that while mPFC unit firing rates did not differ across correct and incorrect trial performance, theta entrainment of mPFC units was absent during error trials. Seemingly, therefore, it is not the mPFC activity *per se*, but the coupling of it with theta oscillatory activity in the HPC that determined accurate performance on this delayed non-match to sample task (Hyman et al., 2010).

Anatomical connections between the HPC to the mPFC that might support these processes are well documented (Jay and Witter, 1991; Vertes, 2006; Parent et al., 2009). Unidirectional monosynaptic connections link efferent projections from the ventral HPC to the mPFC (Jay and Witter, 1991; Cenquizca and Swanson, 2007; Parent et al., 2009; Fanselow and Dong, 2010) with no direct return projections from the mPFC to the ventral HPC (Vertes, 2006). In contrast, only indirect pathways link the dorsal HPC and the mPFC. Communication between these regions occurs via intermediary brain structures including the entorhinal cortex, peri—and postrhinal cortices, and the nucleus reuniens of the thalamus (Jay and Witter, 1991; Vertes, 2006; Morales et al., 2007; Vertes et al., 2007; Fuster, 2008; Fanselow and Dong, 2010; Varela et al., 2013). In particular, recent work suggests that the nucleus reuniens may have a major involvement in the coordination of activity between these structures (Hoover and Vertes, 2012; Varela et al., 2013).

Communication between prefrontal cortex and hippocampus is clearly important during decision-making. Since schizophrenia is associated with anatomical and functional disruptions in both of these regions one might predict that long-range communication between these areas will also be disrupted (Heckers et al., 1999; Meyer-Lindenberg et al., 2005; Zhou et al., 2008; Benetti et al., 2009; Qiu et al., 2010). In fact, impaired functional coupling has been shown between the PFC and HPC during a working memory task in individuals with schizophrenia (Meyer-Lindenberg et al., 2005) and has been associated with a common genetic schizophrenia-associated variant, in a dose-dependent manner (Esslinger et al., 2009). It was of interest, therefore to determine, whether there was any evidence of disrupted communication between these structures in the MIA model.

Findings from our lab showed that *in utero* exposure to MIA resulted in the reduction of long-range coherence between the mPFC and dorsal HPC in adult offspring during a basic foraging task. Specifically, we demonstrated a significant reduction in coherence in the delta to low-gamma (2–48 Hz) frequency ranges between these two key regions. The gamma frequency disruption noted here is of particular interest as changes in this band have been associated with schizophrenia in many studies. For example, reduced gamma synchrony has been associated with poorer perception (Kwon et al., 1999; Spencer et al., 2003; Spencer, 2008; Spencer and Niznikiewicz, 2008), working memory performance (Basar-Eroglu et al., 2007; Haenschel et al., 2009), and cognitive control (Cho et al., 2006). This finding was the first evidence of aberrant long-range synchrony between the mPFC and the HPC in an environmental risk model of schizophrenia (Dickerson et al., 2010). Further, through the use of *in vivo* single-unit and LFP recordings, MIA animals showed a deficit in the timing and phase of firing of single-unit activity in the mPFC to both local (mPFC-generated), and long-range (HPC-generated), theta and gamma oscillations.

Long-range synchrony was disrupted in the absence of changes in local neural network functioning, as indicated by local power spectral measures, which were not impaired in any frequency range. Further, these reductions were not explained by changes in basic firing properties, gross motor behaviors, or the learning patterns and cognitive demands that might be associated with a more complex behavioral task. This suggests, therefore, that even during a basic foraging paradigm requiring minimal cognitive load, MIA animals exhibited a basal impairment in the ability of neural networks to temporally coordinate their output.

A significant decrease in the PPI measure of sensorimotor gating was also evident in the MIA group when compared to controls, replicating several previous findings from our lab group and others (e.g., Meyer et al., 2005; Wolff and Bilkey, 2010). Our data demonstrated that there was a significant correlation between this behavioral PPI deficit and the aberrant low gamma coherence between the mPFC and dorsal HPC. This was, to the best of our knowledge, the first time an association between this benchmark schizophrenia-like behavioral deficit and dysfunctional coherence had been made. At around the same time Sigurdsson et al. (2010) showed that similar effects were observed in a genetic mouse model (Df(16)A ±) of schizophrenia. They reported a marked decrease in long-range synchrony between the dorsal HPC and mPFC in the delta and theta frequency bands during a working memory task. Our results complement these findings well and point to the potential for genetic and environmental risk factors to produce effects through some common pathway.

Despite a wealth of research, schizophrenia remains a poorly treated disorder. Although the severity of cognitive deficits in schizophrenia are a significant predictor of quality of life for patients (Van Winkel et al., 2007; Tandon et al., 2009), by and large, currently available pharmacotherapy does little to alleviate this dysfunction. CLZ, an atypical antipsychotic drug, is argued to be the most effective antipsychotic currently available in treating cognitive deficits (e.g., Leucht et al., 2009). If aberrant synchrony contributes to the cognitive deficits, then CLZ's ability to treat cognitive symptoms may be due to its impact on oscillatory activity. We therefore investigated whether the deficits in synchrony identified (Dickerson et al., 2010) could be ameliorated through currently available antipsychotic medication. Specifically, we investigated the impact of CLZ on the synchrony of neural networks in MIA animals in order to see if the modulation of synchrony might possibly underlie some of its therapeutic effects.

Our analyses focused on theta frequency changes induced by CLZ due to the evidence for low frequency EEG changes following both acute and chronic CLZ exposure within the human schizophrenia literature (e.g., Lacroix et al., 1995; Roubicek and Major, 1977). This study replicated the reduced synchrony observed between the mPFC and the dorsal HPC in MIA animals while the primary finding was that acute CLZ exposure enhanced long-range theta coherence in a dose-dependent manner (Dickerson et al., 2012). This dose-dependent increase was also seen in local theta power in the mPFC, but was absent in the dorsal HPC. The specificity of this increase to the mPFC may suggest that CLZ exerts its ameliorating effects on synchrony through changes in network activity in the mPFC (Dickerson et al., 2012). Such mechanisms might include CLZ's action at D1 receptors (Lahti et al., 1993), fine-tuning the firing patterns of mPFC pyramidal neurons and increasing the signal-to-noise ratio of these cells (O'Donnell, 2003). It may also involve CLZ's action at 5-HT receptors (Kapur et al., 1999; Miyamoto et al., 2005), and either 5-HT or dopamine, or both, may exert downstream influences on GABAergic interneuron activity which may in turn modulate oscillatory activity. The therapeutic efficacy of CLZ on schizophrenia symptomatology, and notably on cognitive functioning in schizophrenia patients, may therefore be induced through the normalization of both local and long-range synchrony within the theta frequency band.

## Sensorimotor gating

As described previously, PPI is a commonly used measure of sensorimotor gating and is determined by measuring the ability of a weak pre-stimulus to reduce the startle response to a loud acoustic stimulus (Braff et al., 2001a). This attenuation of the startle response appears to be due to a capture of information processing mechanisms by the pre-pulse (Braff et al., 2001a) and the subsequent suppression of input occurring over the next several tens of milliseconds. Individuals with schizophrenia have consistently been shown to have deficits in PPI, showing reduced inhibition of startle in the presence of the pre-pulse (Braff et al., 1978, 2001a; Parwani et al., 2000; Geyer et al., 2001; Ludewig et al., 2003; Meincke et al., 2004). This deficit is thought to underlie the deficits in sustained attention, and the inability to automatically filter, or ‘gate,’ irrelevant thoughts and sensory stimuli from intruding into conscious awareness, that occur in schizophrenia (Swerdlow and Geyer, 1998; Braff et al., 2001b).

We had previously shown that there was a relationship between aberrant long-range coherence, as recorded in a free-foraging situation, and disrupted PPI, with reduced synchrony between the mPFC and dorsal HPC in the low gamma band associated with poorer PPI (Dickerson et al., 2010). Both the mPFC and the HPC have been implicated in the complex circuitry that acts to regulate PPI of the acoustic startle response (Koch and Schnitzler, 1997; Swerdlow et al., 2001), and so we hypothesized that synchrony between the mPFC and the dorsal HPC *during* the PPI paradigm, particularly at the time of prepulse presentation, and particularly in the low gamma band, would be associated with the magnitude of PPI (Dickerson, 2013).

For the group of animals (control *n* = 11; MIA *n* =10) tested in this study there was no significant difference in PPI between the MIA and control group, however there was a significant reduction in long-range synchrony in the MIA group compared to controls (across delta to low-gamma frequency ranges). The absence of a PPI deficit in this cohort likely reflects the subtlety of the MIA manipulation, and may be due to a lower degree of MIA in these litters. The presence of reduced long-range synchrony in the MIA animals suggests, however, that synchrony processes may be more sensitive to immune activation than PPI. In the absence of behavioral deficits in the MIA animals, therefore, data describing the amplitude and power of local EEG activity and coherence between regions were collapsed across all animals (MIA and control).

Our hypothesis was not supported by the data, as we found no evidence of an increase in coherence during the pre-pulse period. Furthermore, there was no significant relationship between PPI and low-gamma coherence, as measured by wavelet coherence techniques, irrespective of whether coherence was assessed during the pre-pulse period or during the no-stimulus baseline condition just prior to the pre-pulse. There was, however, a significant positive relationship between PPI and mPFC-HPC coherence during the startle period. That is, greater gamma coherence during the startle stimulus presentation was associated with increased inhibition of the behavioral startle response. Notably, this relationship was also present during startle-only trials, with low-gamma coherence during (but not prior to) the startle period of startle-only trials positively correlated with % PPI separately determined during 80 dBA pre-pulse + startle trials. Thus, irrespective of whether the pre-pulse was present or not, greater low-gamma coherence during the startle presentation was associated with greater inhibition of the startle response.

One interpretation of these findings is that the degree of gamma-frequency coherence elicited during the startle period reflects the general state of the circuitry regulating the inhibition of the startle. Thus, high coherence during this period may indicate the rapid and full engagement of all potential gating systems while poor coherence during startle may reflect the opposite state. The question of why these relationships weren't evident in the baseline or pre-pulse periods may simply result from there being an insufficient stimulus available during these periods to fully activate coherence-based neural systems. In this regard it must be remembered that although the previous findings, showing a relationship between coherence and PPI, were based on coherence recordings conducted in a foraging situation where there is no clear initiating signal (startle or prepulse), they were the result of an analysis of continuous data recorded over a total period of around 100 min per animal. In contrast, for the current experiments, data acquisition was limited to blocks of around 120 ms, across multiple trials totaling around 1.2 s per time-period per animal. Signal averaging was, therefore, considerably constrained in the latter experiment and so the opportunity to determine coherence events may have been limited to situations where a larger synchronized neural response was initiated (the startle).

Although our primary concern in this study was to examine effects in the low gamma frequency band, we also noted that a distinctive phase-reset occurred in the theta band EEG in response to the pre-pulse presentation. To examine this effect we conducted a wavelet-based analysis of theta-frequency EEG recorded from the mPFC and dorsal HPC. This revealed a significant increase in peak coherence during the pre-pulse period compared to the baseline immediately prior. Although theta coherence during the baseline, pre-pulse or startle period was not correlated with % PPI, it was negatively correlated with startle magnitude. Coherence during the equivalent period on interleaved startle-only trials (where no pre-pulse was presented) was not found to be associated with startle behavior. This indicates that the pre-pulse triggers an increase in coherence at theta frequency, which in turn influences the startle response. That this relationship was not also found with % PPI suggests that the theta coherence present during the pre-pulse period is related to the behavioral response specifically, as opposed to the mechanisms that allow for inhibitory modulation of the startle response. The fact that coherence effects only become apparent during the pre-pulse period may be because an initiating and synchronizing signal is required in order to push these signals above some noise floor.

In sum, these data demonstrate that the MIA intervention is capable of modeling many of the features of schizophrenia pathology, including disruptions in synchrony. It provides, therefore, a useful platform from which to investigate both the mechanisms underlying, and the ontogeny of, these aberrant synchronous processes. Importantly, the model also generates fundamental and translatable behavioral deficits, such as reduced PPI, that allow for the relationships between neural and behavioral processes to be explored. Our data, as described here, indicate that a systemic deficit in coherence between mPFC and HPC (and possibly other regions) is associated with poor PPI performance. This deficit is not, however, specific to responses to the pre-pulse stimulus, but rather may reflect a more general, tonic deficit in modulatory tone. It remains to be seen what mechanisms underlie this effect, however, if mPFC-HPC connectivity is important, then recent work that has focused on links between these structures should be noted. This work has identified neurons in the nucleus reuniens of the thalamus that both receive projections from mPFC and project to the HPC (Vertes et al., 2007; Varela et al., 2013). It has been suggested that these cells might be critical for the synchronization of activity in the hippocampus and the prefrontal cortex during exploration or in the consolidation of memory. This proposition is supported by recent findings showing that inactivation of the ventral thalamus, including the nucleus reuniens, resulted in impaired strategy shifting in a task that required both HPC and mPFC involvement (Cholvin et al., 2013). Furthermore, optogenetic activation or suppression of reuniens neurons modulated the generalization of fear memories (Xu and Südhof, 2013). Further exploration of this connectivity and its possible role in schizophrenia (Zhang et al., 2012) using the MIA model, will provide insight into critical mechanisms driving dysfunctional information processing which, in turn, underlie some of the deficits in sensory and cognitive functioning in schizophrenia (Braff et al., 1978; Perry and Braff, 1994). A better understanding of these deficits will in turn lead to better targeting of interventions, for example, through modulation of neurotransmitter systems known to play key roles in synchronous oscillatory activity. These include both NMDA receptor-glutamate (Homayoun and Moghaddam, 2007), and GABAergic systems (Abi-Dargham and Moore, 2003), (Lewis et al., 2005; Zhang et al., 2012), selective modulation of which may facilitate the more efficient information processes necessary for effective higher-order cognitive functioning.

### Conflict of interest statement

The authors declare that the research was conducted in the absence of any commercial or financial relationships that could be construed as a potential conflict of interest.
